# Model Test of Anchoring Effect on Zonal Disintegration in Deep Surrounding Rock Masses

**DOI:** 10.1155/2013/935148

**Published:** 2013-08-12

**Authors:** Xu-Guang Chen, Qiang-Yong Zhang, Yuan Wang, De-Jun Liu, Ning Zhang

**Affiliations:** ^1^Institute of Tunnel and Urban Railway Engineering, Hohai University, Nanjing 210098, Key Laboratory of Ministry of Education for Geomechanics and Embankment Engineering, Hohai Univ., Nanjing 210098, China; ^2^State Key Laboratory for GeoMechanics and Deep Underground Engineering, China University of Mining & Technology, Xuzhou 221000, China; ^3^Research Center of Geotechnical and Structural Engineering, Shandong University, Jinan 250061, China

## Abstract

The deep rock masses show a different mechanical behavior compared with the shallow rock masses. They are classified into alternating fractured and intact zones during the excavation, which is known as zonal disintegration. Such phenomenon is a great disaster and will induce the different excavation and anchoring methodology. In this study, a 3D geomechanics model test was conducted to research the anchoring effect of zonal disintegration. The model was constructed with anchoring in a half and nonanchoring in the other half, to compare with each other. The optical extensometer and optical sensor were adopted to measure the displacement and strain changing law in the model test. The displacement laws of the deep surrounding rocks were obtained and found to be nonmonotonic versus the distance to the periphery. Zonal disintegration occurs in the area without anchoring and did not occur in the model under anchoring condition. By contrasting the phenomenon, the anchor effect of restraining zonal disintegration was revealed. And the formation condition of zonal disintegration was decided. In the procedure of tunnel excavation, the anchor strain was found to be alternation in tension and compression. It indicates that anchor will show the nonmonotonic law during suppressing the zonal disintegration.

## 1. Introduction

Shallow-buried resources have been decreasing with the rapid progress in global economy. Thus, the exploitation of deeply buried resources has drawn interest from a number of countries. South Africa, Russia, India, and China have recently conducted a series of exploitations of deep mines with embedded depths of more than 1,000 m. In China, the Jinchuan nickel mine, Tongling copper mine, and Dingji coal mine are more than 1,000 m deep, whereas the Jinping II Hydropower Station is 2,600 m deep. The Kolar gold mine in India is 2,400 m deep, and the deepest gold mine worldwide residing in South Africa is 3,700 m deep. This series of new phenomena in underground engineering with increasing embedded depth has caused the emergence of a failure phenomenon called zonal disintegration. Shemyakin et al. [1] defined zonal disintegration as the alternated regions of fractured and relatively intact rock masses appearing around or in front of the working slope during the excavation of tunnels in the deep rock mass. This phenomenon has not been observed in shallow rock engineering. Moreover, zonal disintegration presents a serious hazard to the stability of surrounding rocks (Qian) [2]. 

Zonal disintegration in many deep tunnels has been monitored using the physical probe method. Adams and Jager [3] were the first to observe such phenomenon by using a bore periscope at an embedded depth of 2,000 m to 3,000 m in the Witwatersrand gold mine in South Africa. He reported that zonal disintegration occurred when the tunnel was excavated either by drilling and blasting or the mechanized method. However, explosion was eliminated as the result of zonal disintegration after Shemyakin [1, 4–6] explored zonal disintegration in Taimyrskii deep mine in Russia by using a resistivity meter. 

Zonal disintegration phenomenon differs from the engineering response in shallow rock excavation and as such cannot be explained perfectly under the framework of traditional rock theory. In accordance with the concepts of traditional continuum mechanics, the enclosing rock mass around a deep tunnel is divided into fractured, plastic, and elastic regions from the periphery of the tunnel to infinity. Zonal disintegration, a characteristic of deep rock masses, has been the focus of recent investigations.

**Figure 1:**
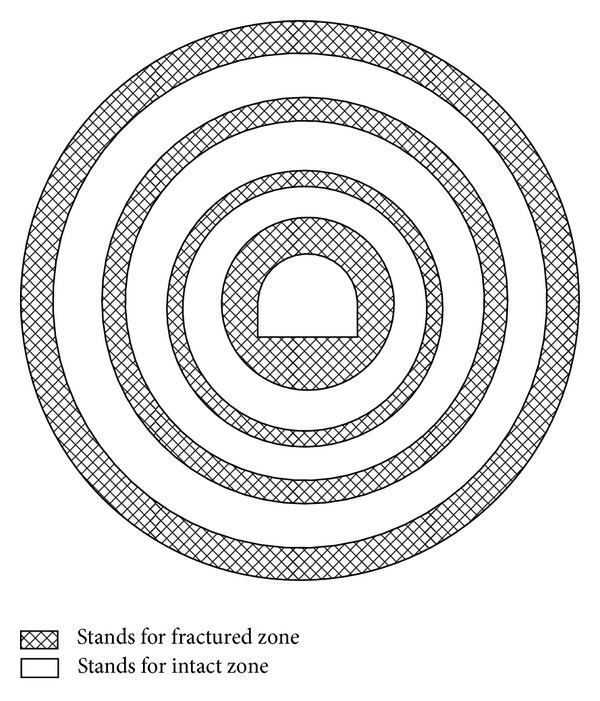
Sketch of the zonal disintegration phenomena in deep tunnel.

A number of experts have used various methods to explain zonal disintegration. Sellers and Klerck [7] indicated that the discontinued surface could be one of the derivations of zonal disintegration. Malan and Spottiswoode [8] analyzed the relationship between the shock bump and the zonal disintegration of a top plate in the surrounding rocks of a mining field. Zhou et al. [9] investigated the dynamic excavation of a deep tunnel to determine the residual strength and the forming time of fractured zones. Gu et al. [10] conducted a compression test on cylinder specimen and regarded axial stress as an important factor for zonal disintegration. Other studies on zonal disintegration have applied different techniques such as a series of compression tests Pan [11, 12], nonequilibrium thermodynamics (Metlov et al.) [13], Hamiltonian time-domain variation (Li et al.) [14], and the non-Euclidean model (Guzev and Paroshin) [15]. In addition, some elastic-plastic theories have been adopted to analyze the forming mechanism of zonal disintegration (Wang et al. [16, 17]; He et al., [18]; Zhou et al. [19–24]; Reva and Tropp, [25]; Tan et al., [26]; Wu et al., [27]; Odintsev, [28]). A zonal disintegration phenomenon is shown in Figure 1.

Zonal disintegration is a unique failure phenomenon posing a large-scale disaster during excavation of deep rock masses (Laptev and Potekhin) [29]. It threatens the stability of deep tunnel and will cause large collapse of rock mass which induces a great loss. It is of great importance to know the anchoring effect on zonal disintegration and the mechanical behavior under anchoring condition in deep rock masses, for the stability of deep tunnel. To the authors' knowledge, anchoring effect on zonal disintegration phenomenon in deep rock masses is not investigated previously. 

In this paper, the Huainan coal mine in which zonal disintegration occurs in China was taken as the engineering background. The model tests on zonal disintegration were carried on in the condition of anchoring and without anchoring, in separate. The model was built using an independently developed barites-iron-sand cementation analogical (BISA) material. Through the analogical model test, the damage pattern with and without anchoring was observed. The nonlinear deformation changing laws were clarified by using a precise optical apparatus. Based on this, the anchoring effect and forming condition of zonal disintegration in deep rock masses is revealed.

## 2. Similarity Theory and Analogical Material

The geomechanical model test is an important scientific research method. Similar to prototype engineering, the model was designed based on the similarity principle. An optical measuring apparatus was used in the geomechanical model test. The stress and displacement changing rules of the model and strain of the anchor were monitored to determine the deformation laws of prototype engineering. The model test exhibits an advantage in studying the failure mechanism of underground cavities over in situ observation, which relies on auditory-visual perception and is time-consuming. 

The geomechanical model test is an effective reduced scale method used for investigating special engineering problems based on the similarity principle. The changing laws of stress, strain, and displacement can be monitored by designing the model similar to that of prototype engineering. Following the similarity principle, the data observed from the model test can be used to reveal the stress distribution laws and the mechanism in prototype engineering, thereby solving actual problems. 

### 2.1. Similarity Theory

The geomechanical model test requires a suitable similar material that can reflect the mechanical behavior of a rock type. The similar material and its prototype must comply with the similarity principle. The theory requires several similarity coefficients defined as ratios of prototype parameters to model parameters to be constant (Fumagalli) [28, 30]:

(1a) $$\begin{array}{c} C_{\sigma }=C_{\gamma }C_{L}, \end{array}$$

(1b) $$\begin{array}{c} C_{\delta }=C_{\varepsilon }C_{L}, \end{array}$$

(1c) $$\begin{array}{c} C_{\sigma }=C_{\varepsilon }C_{E}, \end{array}$$

(1d) $$\begin{array}{c} C_{\varepsilon }=1,\;\;C_{f}=1, \end{array}$$

where *C*_*σ*_, *C*_*ε*_, *C*_*E*_, *C*_*L*_, *C*_*γ*_, *C*_*f*_, *C*_*ϕ*_, and *C*_*μ*_ indicate the similarity ratios of stress, strain, MOE, geometry, volume weight, COF, internal friction angle, and passion ratio, respectively.

The analogical material should have the properties of high volume-weight, low deformation module, and changeable inner friction angle. No crude material can fulfill all these demands, and thus, the similar material should be assembled artificially. According to the similarity theory, the mechanical parameters of the model can be readily obtained through the prototype.

### 2.2. Proportion of the Similar Material

There are several Institutes researching on the similar material, such as ISMES (Institute of Experimental Models and Structures) in Italy, LNEC (National Laboratory for Civil Engineering) in Portugal, and Tsinghua University in China [30, 31]. Their work shows that whether the model test can reflect the prototype engineering's mechanical response depends on the chosen materials. The suitable material should reflect the mechanical behavior of prototype engineering. The proportion of each component is important for model simulation.

**Figure 2:**
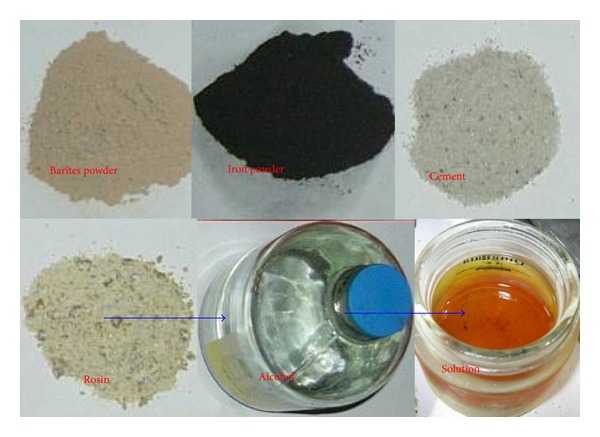
Proportion of the analogical material. The barites powder, iron powder and quarts sand are mixed together to make the aggregate; the solution of rosin and alcohol make the glue.

**Figure 3:**
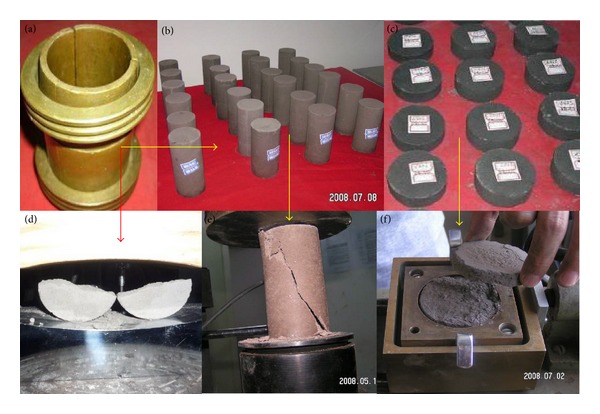
Mechanical test on analogical material. (a) is the specimen mould. (b) is the specimens for UCS. (c) is the specimens for direct shear test. (d) is the Brazilian test. (e) is the uniaxial test. (f) is the direct shear test.

The barite powder, iron powder, and quartz sand are selected to form the aggregate, whereas the alcoholic solution of rosin is used as the mucilage glue (Figure 2). The proportion of the aggregates and the concentration of the alcohol solution of rosin decide the mechanical behavior of the BISA material. The barite-iron-sand (BISA) material was developed through hundreds of groups of proportioning tests. The specimens of similar material were built by pouring the material into a mould and compressing it (Figure 3). The material exhibits the following advantages: stability in performance, widely variable mechanical parameters, low price, high volume-weight, easy processing, and no toxicity or side effects. The BISA material, which can be used for modeling a tunnel or underground powerhouse, has obtained a patent in China. The material can be used to simulate all kinds of rocks, including hard and soft rocks. The proportion of the material composition for surrounding rocks in the Dingji coal mine was determined via the physical-mechanical parameters test on the analogical material. The mechanical parameters of the material were tested in the proportion of each component in the material (Figure 3). 

The laws between the mechanical parameters of material and components proportion were derived by mechanical testing on hundreds of specimens. 

The medium sandstone in the bed stratum from the Dingji coal mine and the Huainan mining area was processed into a specimen. The physical-mechanical parameters of equivalent anchors were tested. The mechanical parameters of the medium sandstone consist of the following: unconstrained compressive strength (UCS) of 88.55 MPa, tensile strength of 14.01 Mpa, and deformation modulus of elasticity (Edef) of 12.97 GPa. The similarity ratio of volume weight for the analogical material is set to 1 : 1, whereas the similarity ratio of geostress is set to 1 : 50. Thus, according to the similarity principle, the mechanical parameters of the prototype and similar material are as follows (Table 1).

According to the curves between the mechanical parameters and the material proportion, the proportion of each component for medium sandstone is as follows (Table 2).

### 2.3. Equivalent Anchors

The parameters of anchor adopted in engineering are: *ϕ*20@800 × 800 mm, *L* = 2.2 m. According to the similar principle, the parameters of model anchor can be got from the prototype anchor (Table 3). 

**Figure 4:**
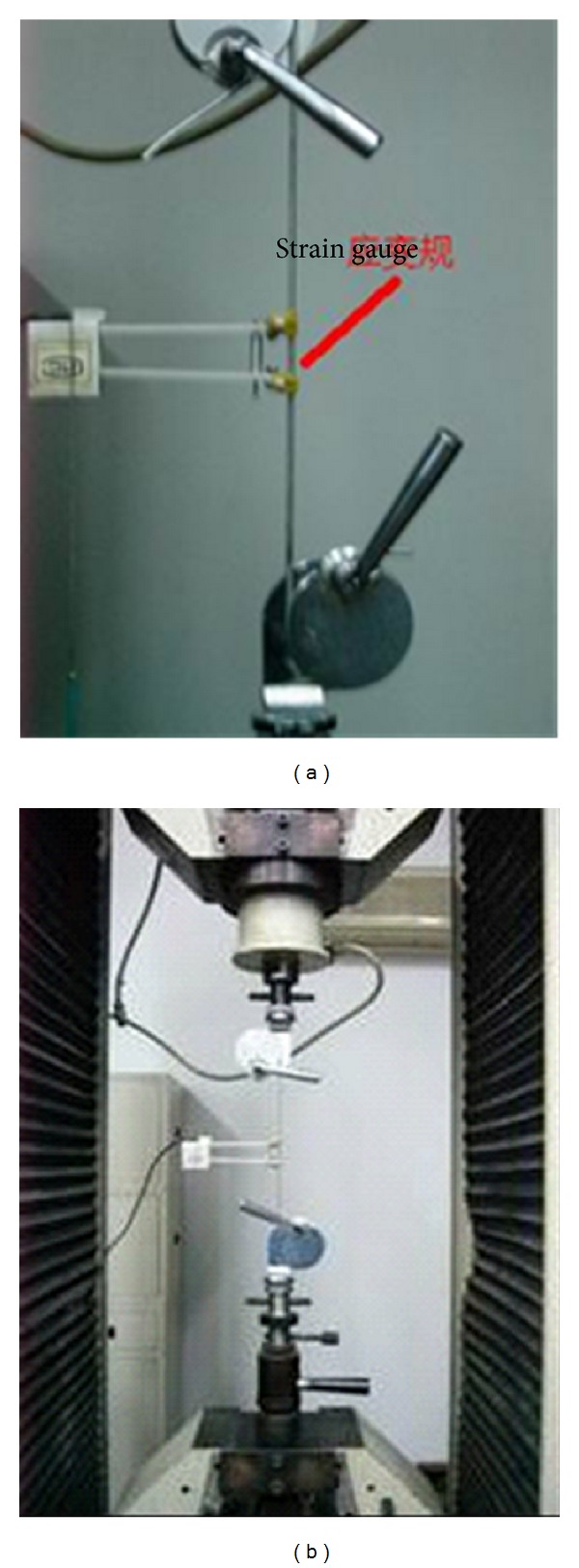
Mechanical test on equivalent anchor.

After the mechanical test on the serious metal materials (Figure 4), the aluminum wire is selected as the equivalent anchor.

## 3. Development of the Model Test System

### 3.1. Development of the Triaxial Model Test System

**Figure 5:**
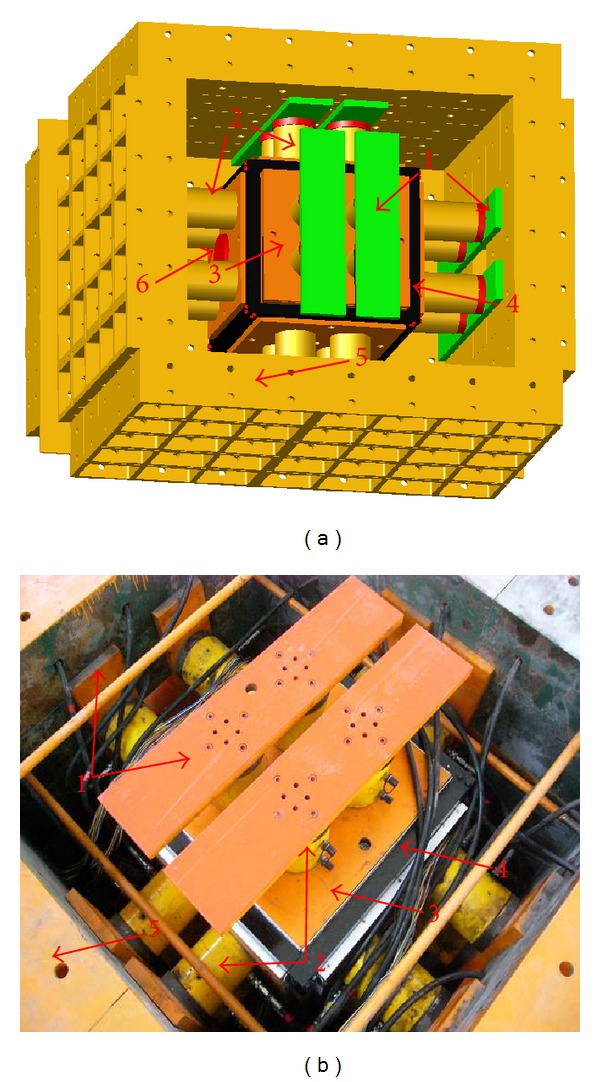
Sketch and photos of the true 3D model test system. The model was set inside the frame, and the platen was set into the frame at 0.5 cm depth. Then, each direction of the model has the reserved displacement of 9 cm. Notes: (1) junction plate, (2) hydraulic jacks, (3) loading platen, (4) oriented frame, and (5) combination reaction frame for loading (6) excavation guiding platen.

In order to simulate the 3 D geostress state of tunnel precisely, the high geostress-triaxial loading model test system was developed independently.  Figure 5 is the design sketch and the photo of the system.

The model test system is comprised of the high geostress loading system, the computerized numerical control (CNC) hydraulic system, and the support reaction apparatus. The high geostress loading system consists of jacks and loading platens, which are used to apply high geostress. It is set inside the support reaction apparatus. One end of the jacks was disposed with loading platen, while the other was fixed to the support reaction apparatus with bolts. There are 6 platens in all, and one contacts each surface of the model, which can apply loading at 3 directions. The dimension of each platen is 0.6 × 0.6 × 0.04 m, which is equal to the size of model surface. There arrange 4 hydraulic jets on each platen, and the capacity of each jack is 40 tons. The loading capacity of each platen is 1600 KN. The high geostress loading system is connected with the CNC hydraulic system. The CNC hydraulic system is used to regulate and control loading value. The support reaction apparatus is composed of box cast steel components, which can be assembled flexibly. It is solid enough to undertake support reaction to 5000 KN high.

During the triaxial compression on 3D model, two adjacent loading platens will interrupt each other because of the volume contraction. So, an oriented frame was developed to solve the problem. The frame consists of 12 steel poles which are connected with each other. Each pole was 5 cm thick and 70 cm long. The model was set inside the frame, and the platen was set into the frame at 0.5 cm depth. Then, each direction of the model has the reserved displacement of 9 cm. A circle platen whose size is similar to the tunnel was fixed into the two platens in front and back of the model. And the platens will be fixed during the loading on the model and taken off when excavation of the tunnel (Figure 5). So, the excavation in 3D model can be solved.

The advantages of the system are shown as follows.

1. High geostress can be applied to the model in all 3 directions independently and synchronously. And the geostress can be remained stable for long term.
2. The problem of two adjacent loading platens that interrupted each other which was caused by the triaxial compression was solved. And the excavation in 3D model was realized.
3. The loading capacity is large enough to 2000 KN, which can simulate the cavern deep to 5000 m. And the loading precision reaches up to 0.5%.

### 3.2. Development of the High Precision Optical Measuring System

**Figure 6:**
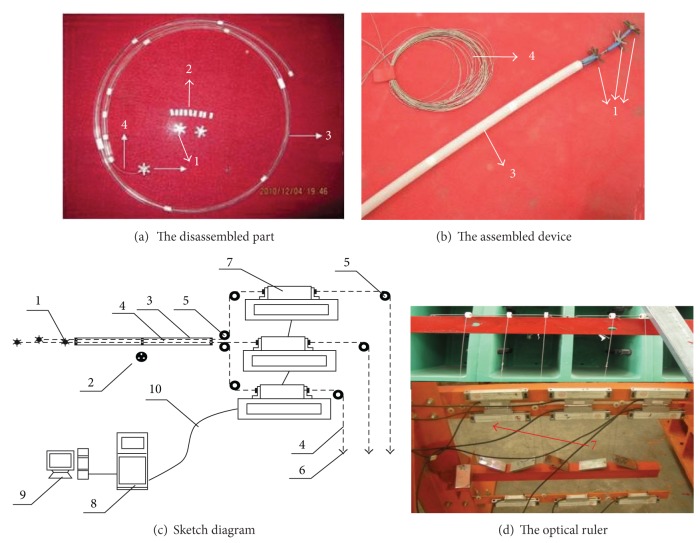
Grating ruler multipoints displacement measuring system (GRDS). (1) Fixed end, (2) guding frame, (3) soft pipe, (4) flexible steel wire, (5) displacement transfer roller, (6) balance weight, (7) optical ruler, (8) STS, (9) DAS, (10) cable.

A micrometer is necessary because the multi-point displacement meters are too huge for the model. Thus, a micrograting ruler multipoint displacement measuring System (GRDS) was developed to monitor the displacement in the rock mass of the model (Figure 6). The Moiré fringe is used to enlarge the displacement in the model. The GRDS is composed of rack, grating rulers, steel wires, balance weight, fixed end, signal translating system (STS), and data acquisition system (DAS). Several fixed ends were buried at the measuring spots inside the model. The fixed ends were connected to the grating ruler outside the model with the steel wire. The steel wire was made up of special sterepsinema without any axial deformation, and thus, it is very flexible and can be bent arbitrarily. The steel wire was wrapped with Teflon pipe to eliminate friction between the steel wire and the model. The GRDS transformed the displacement of the model to the grating ruler through the mechanical method. Then, the real displacement was transferred to the optical signal by using an optical ruler. The STS was connected to the grating ruler with the guide line, and the optical signal was transformed to digital signal and then transferred to the DAS. The displacement data of the model was displayed and stored instantaneously. The high precision (1 *μ*m) GRDS can be used to measure the displacement in any direction as it can be bent at any angle. 

**Figure 7:**
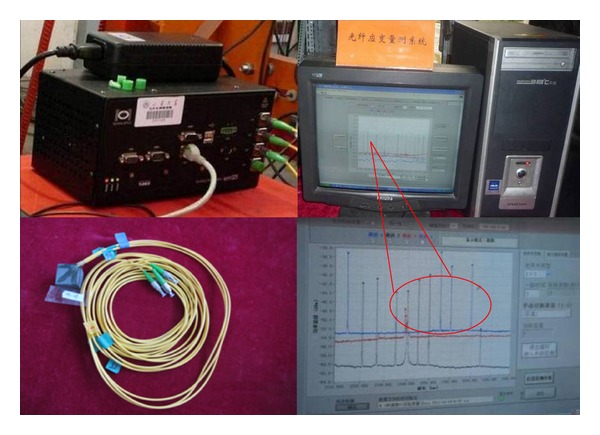
Grating strain sensor measuring system.

The fiber bragg grating (FBG) was stuck to the analogical material blocks to make the grating strain sensor. And it can be used to measure the strain in each direction inside the model (Figure 7).

The FBG can measure the strain changing outside through the centre wavelength mobile. The relationship between the centre wavelength *λ*_*B*_ and the effective refraction index *n*_eff_ of grating and the period of grating Λ is

(2) $$\begin{array}{c} \lambda_{B}=2n_{\text{eff}}\Lambda , \end{array}$$

where *λ*_*B*_ is centre wavelength of the Bragg grating; Λ is the period of grating; *n*_eff_ is the effective refraction index.

When the fiber is stretched, Λ and *n*_eff_ will change, and then the centre wavelength *λ*_*B*_ will drift. The wavelength gets larger when the fiber is stretched, while it gets smaller when the fiber is compressed. The linear relationship will be satisfied:

(3) $$\begin{array}{c} \Delta \lambda_{B}=\lambda_{B}\left(1-P_{e}\right)\varepsilon =K_{e}\varepsilon , \end{array}$$

where, Δ*λ*_*B*_ is the variation of wavelength; *P*_*e*_ is the effective optical coefficient (0.22, generally); *ε* is the axial strain of fiber; *K*_*e*_ is the sensitivity of strain measuring.

When the grating generates strain in the stress condition, the pitch of the grating will change to ΔΛ, which will make the wavelength change to Δ*λ*, so the strain can be got:

(4) $$\begin{array}{c} \varepsilon =\frac{\Delta l}{l}=\frac{\Delta \lambda }{\lambda }. \end{array}$$

## 4. Construction of the Model

To study the anchoring effect on zonal disintegration, a model was built and divided to two halves to make compassion: one half is anchored and the other half is nonanchoring. 

### 4.1. Simulation Range of the Model

The model size is limited by the reasonable size of the steel frame. Within the frame, if the similarity constant for the geometry *C*_*L*_ is smaller than 1 : 50, then the model tunnel will be too small to be excavated. If the *C*_*L*_ is very large, then the relevant monitoring devices will be too difficult to install and the monitoring data will be inaccurate. Considering these prior factors, 100 is taken as the optimal similarity coefficient *C*_*L*_.

The simulation range of the prototype is 30 m × 30 m × 30 m. According to the geometry similar scale 1/50, the dimension of the model is determined to 0.6 m × 0.6 m × 0.6 m and the model tunnel is 100 mm × 77.6 mm.

### 4.2. Flow of Model Construction

**Figure 8:**
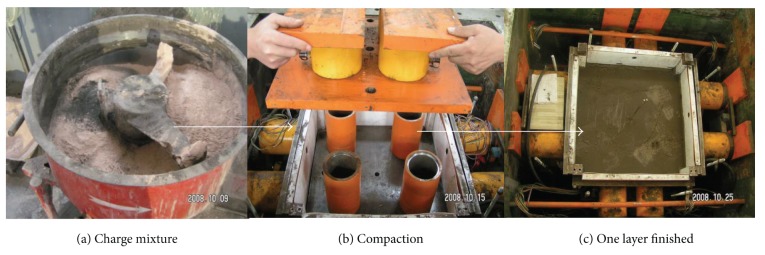
Procedure of the model building.

The model was made delaminating. Each layer is 10 cm high, so 7 times are needed in all to finish the entire model. The measuring components were set up when the material reached the designed height (see Figure 8).

### 4.3. Burying of the Measuring Components

**Figure 9:**
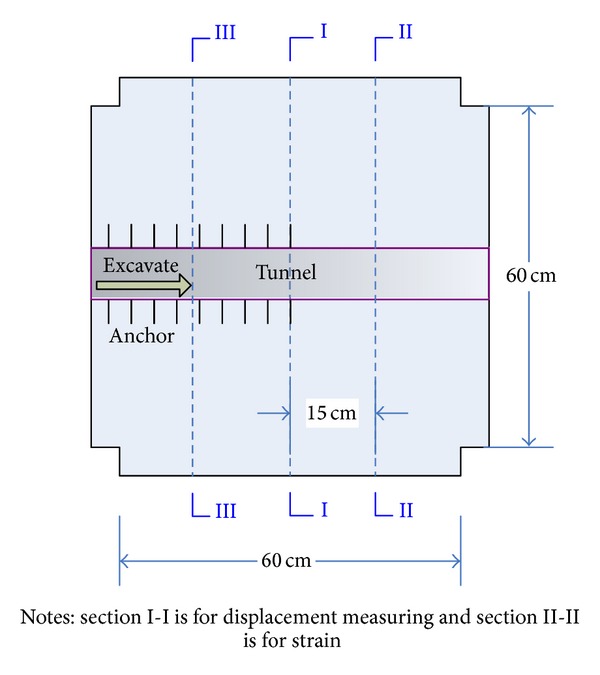
Layout sketch of monitoring sections in the model. The model is divided to two parts: anchored area and the area without anchoring. The measuring sections were set to 3, which divides the model to 4 parts evenly. The II measuring section was chosen to be the middle part of the model, while the I and III sections are quarter and three-quarters of the model. Then, the measuring section can be used to monitor the deformation inside the model.

**Figure 10:**
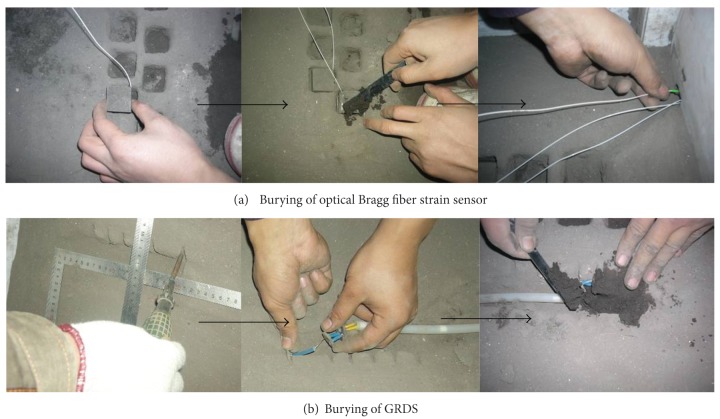
Burying procedure of the optical measurement sensor. The sensor is Fiber Optical Bragg grating sensor, where the precision is 10^−3^ *με*. The spacing between those sensors is 1 cm.

The measuring components were disposed in the model, including the optical multipoint displacement meter and the grating Bragg optical strain sensor, which were used to monitor the displacement and strain changing in the surrounding rocks, respectively. Figures 9–10 show the section sketch and burying procedure of the measuring components, in separate.

### 4.4. Distribution and Burying of the Anchor

**Figure 11:**
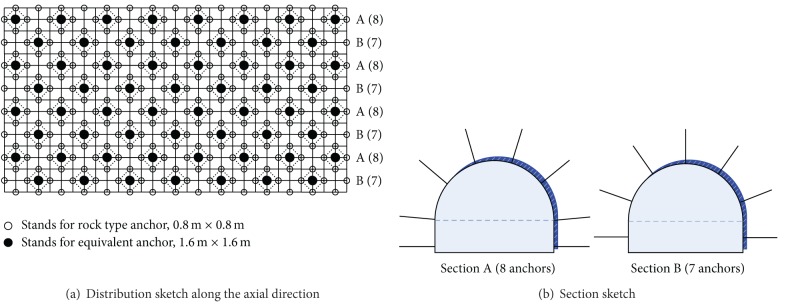
Layout sketch of the equivalent anchor.

The anchored area was applied with anchors, while the other half was not. Because the anchor surrounding the tunnel is too intensive, an equivalent principle of pulling resistance is adopted to dispose the anchor. A thick anchor is replaced by 4 thin anchors. Considering of the quincuncial disposal of anchor, it is not equal between the two adjacent sections. There are 8 anchors in section A and 7 anchors in section B, in separate. The two sections are arranged alternately (Figure 11). The grouting material is mixed by the high thickness alcohol solution of rosin, whose stickiness and fluidity are both well to meet the grouting demand.

1. Arrangement of anchors. Figure 11 is the layout sketch of the equivalent anchor. As the figure shows, the equivalent anchor is arranged according to the quincunx. There are 8 anchors in section A and 7 anchors in section B. The inter-row spacings are both 1.6 m. The two sections are arranged alternately along the axial direction of tunnel.
2. Burying of the anchors. The embedded method and grouting burying method are adopted together. The embedded method is adopted for the anchor of the middle of model. The grouting burying method is adopted for the entrance of the the tunnel.

**Figure 12:**
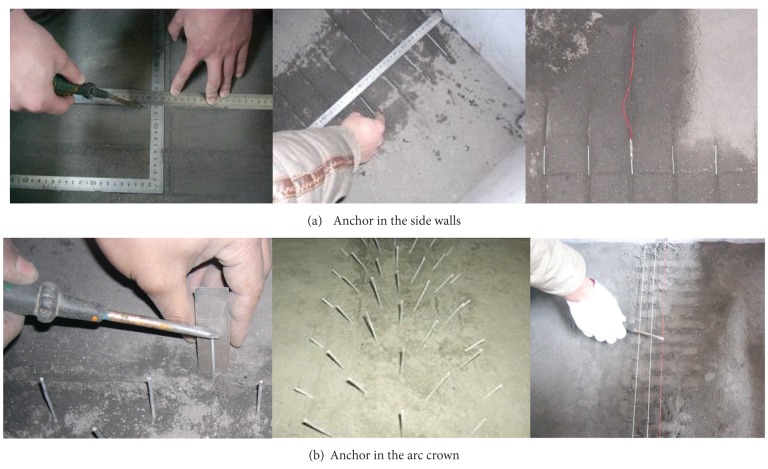
Burying procedure of analogical anchor.

Figure 12 is the burying procedure of the embedded anchor, where (a) is for the side wall and (b) is for the arc crown. After burying, the material is backfilled and tamped, till the completion of the entire model.

The anchor burying of two sections near the cavern was carried during the procedure of tunnel excavation. The details are shown in Figures 14(b)–14(d).

### 4.5. Tunnel Excavation and Model Testing

**Figure 13:**
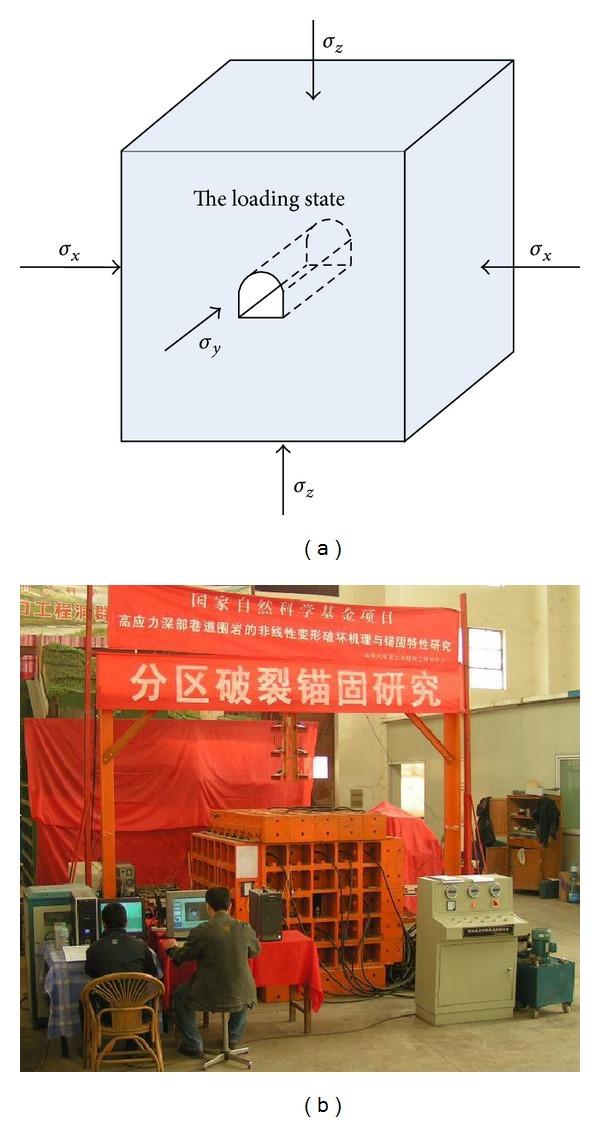
Loading state and test procedure of the model.

The deep tunnel is in the 3D geostress state. In order to simulate the geostress fields accurately, the model was loading in true 3D state (Figure 13). During the excavation, the displacement and strain changing laws were monitored and recorded (Figure 13). 

The self-weight stress is *γh*. The loading which is perpendicular to the tunnel axial is 1.5*γh* (coefficient of horizontal pressure is 1.5). Where *γ* is the volume weight, *h* is the embedded depth of the tunnel. The loading value is listed in Table 4.

**Figure 14:**
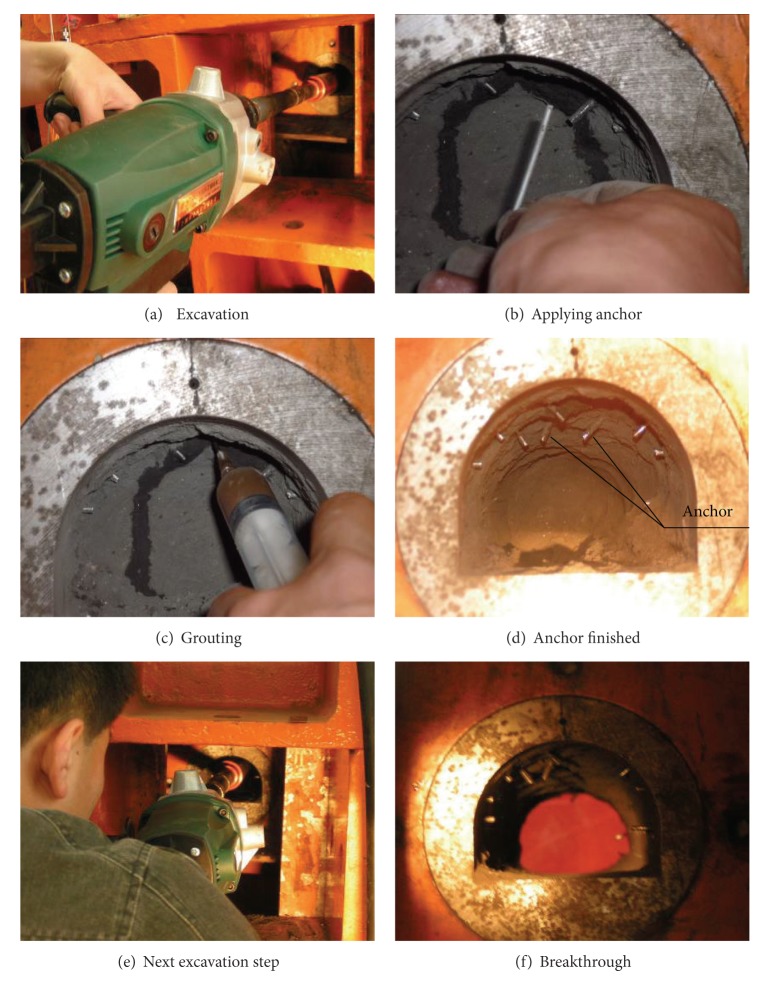
Excavation procedure of model tunnels.

The excavation procedure of the model is shown as follows: (Figure 14).

## 5. Results of Model Test and Discussion

### 5.1. Displacement from the Grating Extensometer

**Figure 15:**
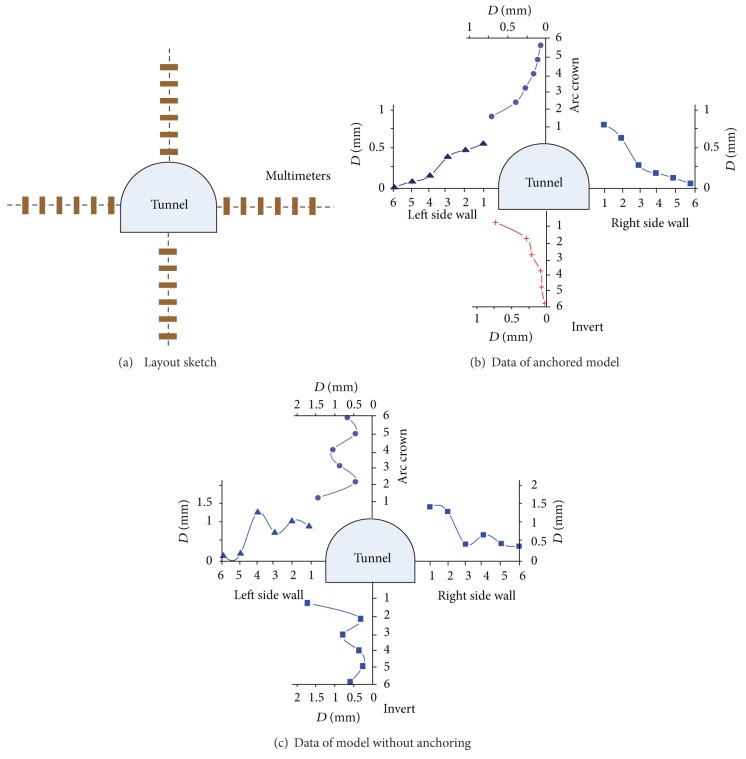
Displacement surrounding the model tunnel.

Figure 15 shows the drawing sketch of grating extensometer. The displacement of surrounding rocks is labeled and connected using the smooth curve after excavation. Then, the changing laws of displacement can be got. The displacement changing sketch is placed together to compare. It is known from the sketch of displacement surrounding the tunnel.

1. The displacement shows a very different changing law between the anchored model and nonanchoring model. In the anchored model, the displacement decreases monotonously as the distance to the tunnel wall increases. It is similar to the shallow embedded tunnel. While in the nonanchoring model, the displacement presents the undulate changing status, wherein the wave crest and the trough are arranged alternately. It completely differs from the shallow embedded tunnel.
2. Compared with the dissembled model, the area of larger displacement is the severely damaged region. In the anchored model, the displacements of no. 1 spot near the periphery are larger than the other area. It indicates that this area is the damage zone in the traditional perception. This is in accordance with the phenomenon of periphery damage seriously during the excavation. The displacement in arc crown is larger than the side walls. This is corresponding to the phenomenon of serious damage and collapse in arc crown. In the model without anchoring, the wave crest region with a larger displacement is the fractured zone, whereas the trough region with a smaller displacement is the intact zone. The additional displacement in the wave crest area, which is caused by the circular fracture, increases the total displacement.

### 5.2. Strains from FBG Strain Sensor

The strain results show that both the radial and tangential strains were negative before the excavation. This indicates that the surrounding rocks were in compressive state. When it is excavated to the strain monitoring section, the radial strain *ε*_*r*_ turns to positive, which indicates that the tensile strain appear in the radial direction. The phenomenon was in accordance with the elastic-plastic strain field analysis of the ideal circular tunnel. 

**Figure 16:**
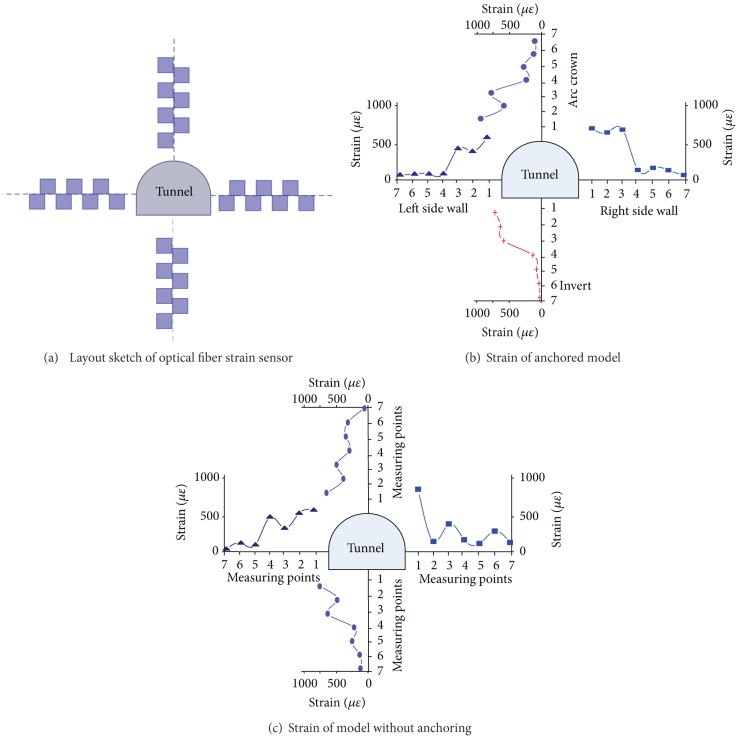
Radial strain around tunnel measured by optical fiber strain sensor.

Figure 16 is the diagram sketch of FBG strain sensor. The radial strain is labeled in the surrounding rocks nearby the tunnel after the tunnel excavation and measurement. 

As is shown in Figure 16, the radial strain in the surrounding rocks both in the anchored model and nonanchoring model shows the same changing law: it presents the fluctuate distribution with the distance increasing from the periphery. The wave crest and the trough distribute alternately. This law is totally different from that in the shallow embedded tunnel. The phenomenon indicates that under the high geostress loading, the radial tensile strain is the highest in a certain area of the surrounding rocks. This area is almost circular and concentrated in the tunnel, and identified as the elastoplastic zone of the surrounding rock.

Zonal disintegration phenomenon does not occur in the side walls and arc crowns of anchored model. It is because the reinforcement effect improves the capacity of fracture-resistance of surrounding rocks. There is no reinforcing effect in the nonanchoring area, such as the invert of anchored half model and the half model without anchoring. Zonal disintegration phenomenon occurs in these areas.

### 5.3. Anchor Strain in the Middle Section of the Model

**Figure 17:**
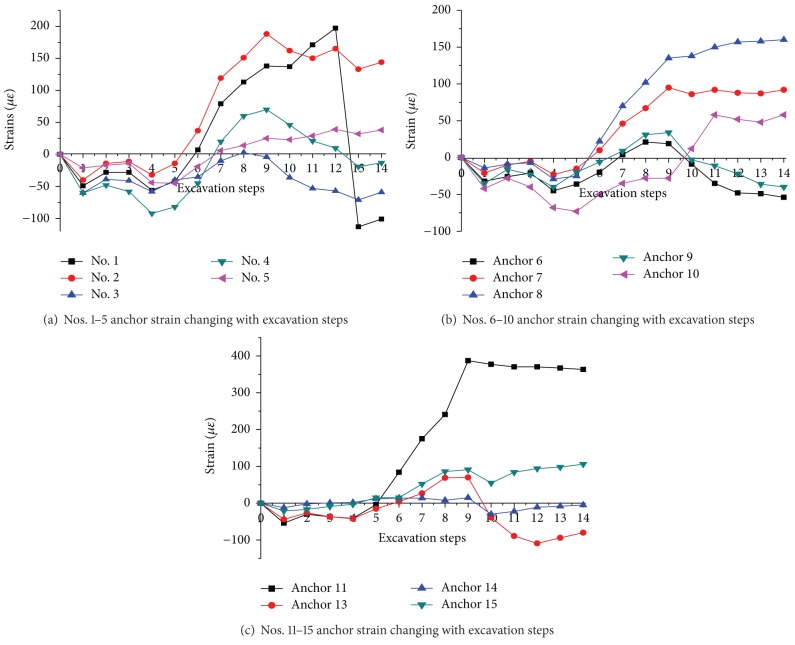
Anchor strain changing with the excavation steps. Note: No. 12 anchor was damaged during the test and the data could not be recorded.

Figure 17 is the changing laws of anchor strain in the middle section of the model measured by the strain gages.

1. The anchor strain is minus at the beginning of tunnel excavation. It indicates that the anchors are in compressive state in high loading condition. It turns to positive at the excavation of step 6 which is near the middle of the model (there are 14 excavation steps in all). It indicates that anchors turn into tensile state and work.
2. Most anchors (except nos. 1, 6, and 11) turn to maximum state at excavation of 7 or 8. This is because the anchor works at the largest effect when excavated in the middle of the model, which is the section of anchor strain measurement.
3. A mount of anchors (nos. 1, 4, 5, 8, 10, 13, and 14) shows the phenomenon of positive and negative alternation during the excavation process. It indicates the tension and compression alternating of anchor, which is first observed in the geomechnical model test.

Fang [32] used to observe the similar special phenomenon of tension and compression alternating in the deep tunnel of Jinchuan nickel mine, 1150 m deep. He believes that it is the self-organized phenomenon and the inherent characteristic of deep tunnel. 

### 5.4. Fracture Shape inside the Model

**Figure 18:**
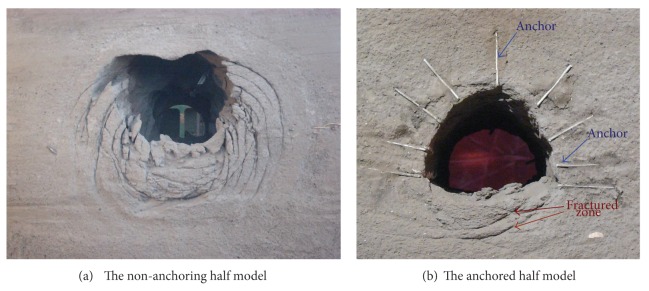
Failure distribution of the surrounding rock mass after tunnel excavation.

Figure 18 is the photos of dissembled model which show the damage pattern. 

The zonal disintegration phenomenon of alternating fractured zone and intact zone occurs in nonanchoring model. There are 4 fractured zones and 4 intact zones arranging in space which are in accordance with the fractured shape of the in-situ observation in DINGJI coal mine. There are 2 obvious fracture lines in the bottom of the anchored model. Zonal disintegration also occurs in the invert of the model. But there is not any fracture line occurs in the arc crown and side walls; that is, zonal disintegration phenomenon did not occur in the anchored part of model. 

After comparing with the model of anchored and nonanchoring, it indicates that the anchoring effect reinforces the anchoring area of model to be a unity. This impact makes zonal disintegration difficult to occur. 

### 5.5. Analysis of the Anchoring Effect on Zonal Disintegration

**Figure 19:**
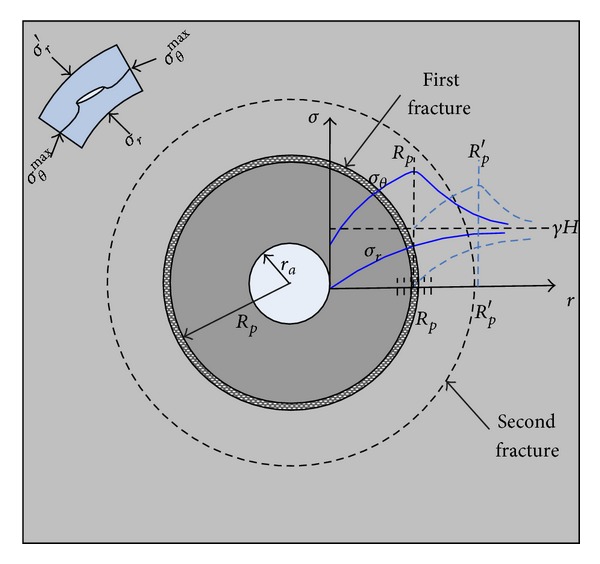
Elastoplastic geostress fields in surrounding rock mass and forming process of zonal disintegration.

Under high geostress conditions, the surrounding rocks near the cavern wall yield to plasticity. The principal stress field in the plastic and the elastic zones is as follows (Figure 19). As is shown, the tangential stress is the maximum principal stress. At the location of *r* = *R*_*p*_, the tangential stress is the summit value. 

According to Griffith's criterion, a fracture occurs when the UCS of rocks under pressure reaches the threshold value. Fairhurst and Cook (1966) [33] indicated that microcracks would initiate and extend in the direction of the maximum principal stress when compressive stress reaches Griffith's strength *σ*_*s*_. This finding explains the longitudinal splitting of the rock specimen and the slabbing of the surrounding rocks in the rectangular openings, which also holds true for the circular cavern. Given a unit rock in the system of the polar coordinates (Figure 19), the second circular fracture extends towards the direction of the maximum principal stress (i.e., the tangential direction) when stress fulfills its relationship with the mechanical parameters of the surrounding rocks. The fracture is expected to transfix and form the circular fracture (i.e., the first fracture of the zonal disintegration) when compressive loading is sufficiently large. Fracture formation causes geostress redistribution in the surrounding rocks, inducing the formation of other ultimate equilibrium plastic zones. The second circular fracture occurs when the summit tangential stress is sufficiently large. Zonal disintegration occurs during the process cycle (Figure 19). 

In the condition of high axial geostress, the surrounding rock mass has the trend of zonal disintegration. When model is anchored, it is reinforced and the threshold value of fracture is enlarged. So, at the same geostress, the zonal disintegration did not occur. The model test indicates the trend of zonal disintegration phenomenon existing under a condition of high axial geostress. And the reinforcement of anchor suppresses the zonal disintegration in the anchored area. During the anchor working, the trend of zonal disintegration which induces it shows the tension and compression alternation. 

## 6. Conclusion

1. Under high axial geostress, zonal disintegration phenomena occur in the nonanchored area, while it did not occur in the fully anchored area. It indicates that anchoring suppresses the zonal disintegration obviously.
2. The radial strain of surrounding rocks displays the fluctuation state where wave crest and rough arrange in interval, both in anchored and nonanchoring area. It indicates that the deep rock masses have the trend of zonal disintegration under the high axial geostress.
3. The tension and compression alternation phenomenon of anchor is observed during the tunnel excavation procedure. This special phenomenon is different from the shallow buried tunnel. It indicates that the trend of zonal disintegration exists in the deep tunnel. The reinforcement of anchor suppresses the occurrence of zonal disintegration. During the work of anchor, it shows the alternation of tension and compression. It indicates that the mechanical behavior of the surrounding rocks is nonmonotonic.

The anchor suppresses the growth of zonal disintegration, so the optimization of parameters and disposal of anchor are of great importance to the stability of deep tunnel. The further study is needed to conduct in the future.

## Figures and Tables

**Table 1:** Physical-mechanical parameters of the prototype rock and model material.

Material type	Volume weight/KN·m^−3^	Edef/MPa	Cohesion/MPa	*φ*	UCS/MPa	TS/MPa	NUXY
Prototype	2.62	12970	10	43	88.55	14.01	0.268
Model	2.62	51.88	0.4	43	3.54	0.56	0.268

**Table 2:** Proportion of the analogical material.

I : B : S	Portion of the gypsum	Concentration of the solution	Portion of the solution
1 : 1.1 : 0.42	2.5%	7.5%	5.0%

**Table 3:** Physical-mechanical parameters of prototype and equivalent anchor.

Anchor	Edef/GPa	TS/MPa	Yield strength/MPa	length/cm
Prototype	210	510	345	220
Model	4.2	10.2	6.90	4.4

**Table 4:** The loading value of the model.

Loading direction	Self-weight stress	Perpendicular to the axial	Parallel to the axial
Loading value/Mpa	0.5	0.75	0.75
Loading direction	Self-weight stress	Perpendicular to the axial	Parallel to the axial
